# Tripartite motif 47 promotes the development of thyroid carcinoma through ADAR ubiquitination

**DOI:** 10.1186/s10020-025-01298-z

**Published:** 2025-07-05

**Authors:** Hongzhou Liu, Xiaodong Hu, Tan Li, Yuhan Wang, Xiaomin Fu

**Affiliations:** 1 Department of Endocrinology, First Hospital of Handan City, Handan, 056002 Hebei Province China; 2 Department of Endocrinology, The First Medical Center, Chinese PLA General Hospital, Beijing, 100853 China; 3 Clinics of Cadre, Department of Outpatient, The First Medical Center, Chinese PLA General Hospital, Beijing, 100853 China

**Keywords:** Thyroid carcinoma, Tripartite motif 47, Adenosine deaminases acting on RNA, Glycogen synthase kinase-3β, Ubiquitination, Phosphorylation

## Abstract

**Background:**

Tripartite motif 47 (TRIM47) plays a vital role in the carcinogenesis and drug resistance of various cancers, whereas the function of TRIM47 in thyroid carcinoma (TC) remains unclear.

**Methods:**

Human study and animal experiments were performed. Mass spectrometry, cellular invasion/metastasis assay, chemo-resistance assay, and ubiquitination evaluation were conducted to investigate the interaction between TRIM47 and adenosine deaminases acting on RNA (ADAR).

**Results:**

TRIM47 expression was increased in human tissues and cell lines of TC. Functional experiments demonstrated that TRIM47 expression enhanced malignant biological behaviors. With mass spectrometry, TRIM47 silencing could significantly decrease the chemo-resistance of TC cells to chemotherapeutic drugs. The interaction between TRIM47 and ADAR was mediated through the ubiquitin–proteasome pathway (UPP), which was approved by RNA interference procedure and co-immunoprecipitation.

**Conclusion:**

Comprehensively, glycogen synthase kinase-3β (GSK-3β)-associated ubiquitination is critical in the TRIM47-ADAR-GSK-3β axis. This study demonstrates that TRIM47 interacted with ADAR to facilitate ADAR protein degradation via ubiquitination and GSK-3β-associated phosphorylation, which serves as a novel therapeutic avenue for TC.

**Graphical Abstract:**

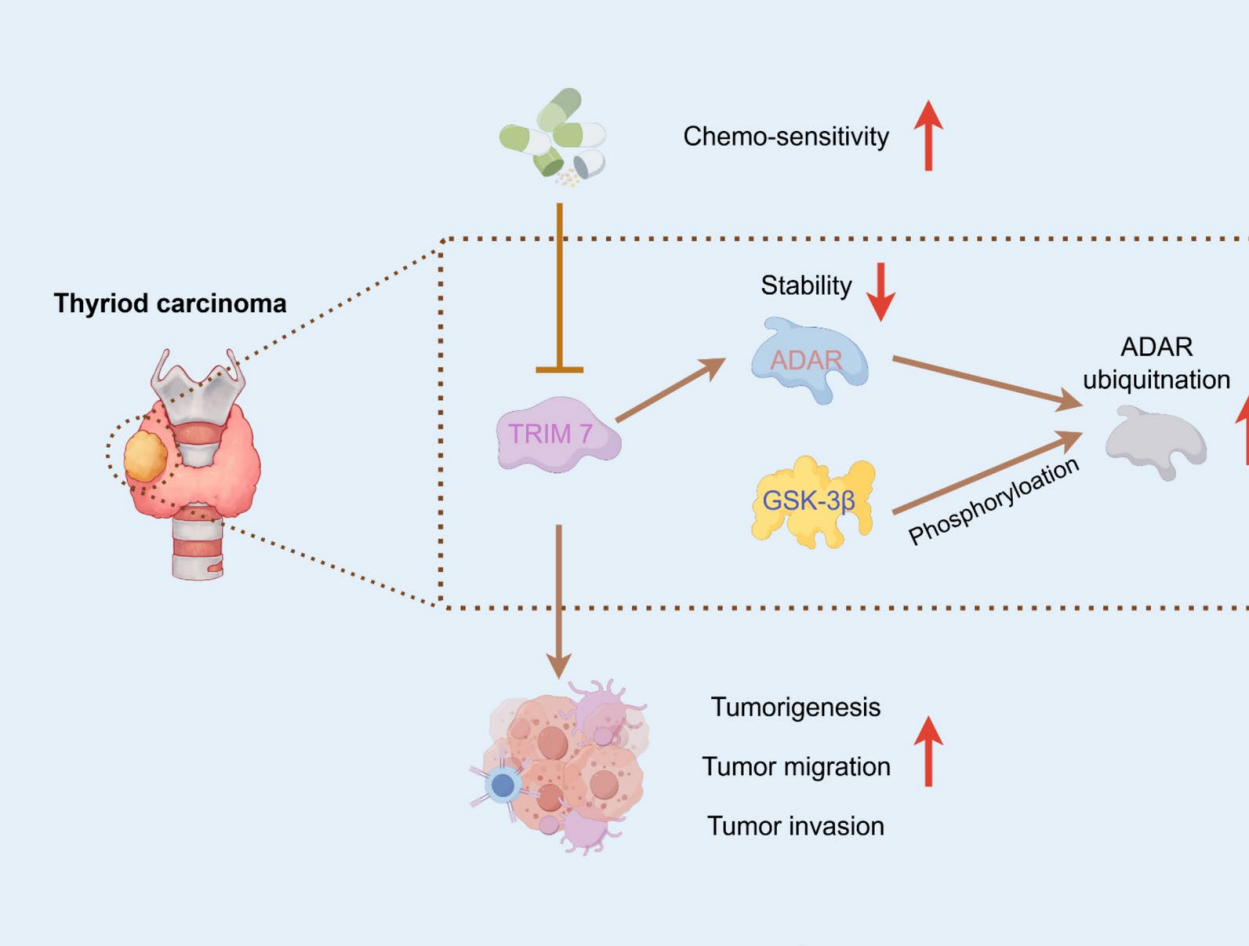

## Background

According to the recent estimates of the American Cancer Society (ASC) in 2020, thyroid cancer (TC) was considered the most rapidly increasing cancer in the United States in the industry countries. Nearly about 44 thousand new cases and over two thousand deaths will be generated in the US (Martinez et al. 2013). The increasing TC cohort has been bringing a huge economic and psychological burden to society and families.

Tripartite motif (TRIM) has been proven to be involved in multiple cellular procedures including cell proliferation, differentiation, apoptosis, cell cycle, and carcinogenesis. Most TRIM proteins affect ubiquitin E3 ligase activity and help post-translational modification (Versteeg et al. 2014). TRIM47 is a member of the TRIM protein family and has E3 ubiquitin ligase activity. It is involved in regulating cell proliferation, migration, apoptosis, and signal transduction pathways in a variety of tumors (Wang et al. 2024). It was reported that TRIM47 activated NF-κB signaling via PKC-ε/PKD3 stabilization and contributed to endocrine therapy resistance in breast cancer (Azuma K et al.,^2021^). TRIM47 promoted head and neck squamous cell carcinoma malignant progression by degrading XAF1 through ubiquitination (Yu C et al., 2025). However, there is no direct study confirming the regulatory role of TRIM47 in TC.

Adenosine deaminases acting on RNA (ADARs) are two-stranded RNA-revising enzymes that can affect RNA and catalyze by converting adenosine (A) to inosine (I) (Roth et al. 2019), and both inosine and guanosine have similar characteristics, this A-to-I editing could apply a great impact on the edited RNA via bypassing genomic information to control nucleotide changes (Ganem et al. 2017). In higher eukaryotes, A-to-I editing is the most featured and usual kind of RNA revising. Originally, ADAR enzyme was considered only on the coding area of certain genes. Subsequently, high-throughput studies figured out that A-to-I editing aims at non-coding sequences as well (Nguyen et al. 2022) and many other characteristics were discovered including its critical function in the microRNA transcriptome (Park et al. 2021). In addition, more studies unveiled that the imbalance of ADAR expression can lead to cancer, amyotrophic lateral sclerosis, and other diseases (Wood 2019), but its pathological role and possible clinical application of ADAR in thyroid cancer are largely unknown. In our study, we focus on the TRIM47 function in TC oncogenesis, and providentially, we found that a critical involvement of ADAR is linked to the accomplishment of TRIM47 through ADAR-associated ubiquitination and the GSK-3β-associated phosphorylation on TRIM47 and ADAR in thyroid cancer oncogenesis.

## Methods

### Human subjects and animal maintenance

All animal care and experiment procedures were conducted following a protocol approved by the Animal Care and Use Committee of our hospital. All human studies were conducted according to the principles of the Declaration of Helsinki, and the study protocol was approved by the Institutional Ethics Committee of Chinese PLA General Hospital (2021–013) with written informed consent from our hospital. Tissues for detecting expression of the proteins in TRIMs and ADARs pathways were collected from the patients. Animal experiments were approved by the Institutional Committee for Animal Research and were performed in conformity with national guidelines for the care and use of laboratory animals.

### Clinical samples

For RNA extraction, the fresh tumor tissue (*n* = 24) and matched non-cancerous tissue (NCT; *n* = 2)) specimens were obtained from the patients who received surgical treatment without prior chemotherapy or radiotherapy in the First Medical Center of Chinese PLA General Hospital, and immediately frozen using liquid nitrogen and subsequently stored at − 80 °C for the following experiments. For immunohistochemistry (IHC), formalin-fixed paraffin-embedded TC and paired NCT specimens were collected from patients undergoing surgery without receiving chemotherapy or radiotherapy. All specimens were confirmed by pathological examinations. Postoperative pathological staging was determined by the tumor node metastasis (TNM) classification of the American Joint Committee on Cancer. All patients were followed up to the preserved timeline or until death.

### Bioinformatics analysis

The RNA seq data of thyroid carcinoma were downloaded from the Cancer Genome Atlas (TCGA) (https://genomecancer.ucsc.edu) and GEPIA databases (http://gepia.cancer-pku.cn/). TRIM47 mRNA expression level was separated by T/N stages. A statistic *p* < 0.05 was recognized as a differentially expressed threshold. Detailed information on the analysis of these open databases has been previously published (Tang et al. 2017).

### Cell culture

Human differentiated thyroid carcinoma (DTC) cell lines (BCPAP, FTD-133) and anaplastic thyroid carcinoma (ATC) cell lines (ATC-1, THJ-16 T, THJ-21 T, THJ-29 T) were purchased from ATCC with certifications of the origin and identity of the cells. The benign human thyroid follicular cell line Nthy-ori 3–1 was obtained kindly from Johns Hopkins University School of Medicine. DTC and ATC cell lines were grown in Dulbecco’s Modified Eagle Medium (DMEM; Sigma, St. Louis, MO, USA), and Nthy-ori 3–1 was maintained in Roswell Park Memorial Institute 1640 (Gibco, BRL, Grand Island, USA) with glutamine. All cell lines were used no later than 6 months after receipt and cultured with 10% fetal bovine serum (FBS; Gibco) and 1% penicillin/streptomycin (Invitrogen, Carlsbad, CA, USA) in an atmosphere of humidified air with 5% CO_2_ at 37 °C.

### Transfection and infection

Cells (THJ-29 T and FTD-133) were seeded in 6‑well plates so that they reached 60–70% confluence on the day of transfection. For plasmid transfections, 2 μg of plasmid DNA was diluted in 250 μL of Opti‑MEM (Invitrogen, Carlsbad, CA) per well. In a separate tube, 4 μL of Lipofectamine 2000 (Invitrogen) was diluted in 250 μL Opti‑MEM and incubated for 5 min at room temperature. The DNA and Lipofectamine 2000 solutions were then combined and incubated for 20 min at room temperature to allow complex formation before adding dropwise to the cells in a serum‑free medium. After 6 h of incubation at 37 °C in a humidified 5% CO₂ atmosphere, the medium was replaced with a complete medium. All constructs were confirmed by DNA sequencing before use. For the generation of stably transfected clones, cells were cultured for 12 days under selection with 200 μg/mL neomycin (G418; Sigma, St. Louis, MO). Single clones were then isolated and expanded for further experiments. Transfection efficiency was assessed by measuring both TRIM47 protein (via Western blot) and mRNA (via RT‑PCR) levels.

### RNA extraction and quantitative real-time PCR (qPCR)

For tissue samples, approximately 50 mg of tissue was homogenized in 1 mL of TRIzol reagent (Invitrogen, Carlsbad, CA) using a mechanical homogenizer. For cell samples, 1 × 10⁶ cells were lysed in 1 mL TRIzol. After incubation for 5 min at room temperature, 200 μL of chloroform was added, the mixture was shaken vigorously for 15 s, incubated for 3 min, and then centrifuged at 12,000 × g for 15 min at 4 °C. The aqueous phase was transferred to a new tube and RNA was precipitated with 500 μL isopropanol for 10 min at room temperature, followed by centrifugation at 12,000 × g for 10 min at 4 °C. The RNA pellet was washed with 75% ethanol, air-dried, and resuspended in RNase-free water. The RNA concentration was adjusted to 1 μg/μL, aliquoted, and stored at − 80 °C. cDNA synthesis was performed using the Reverse Transcription Kit (Promega, Madison, WI, USA) according to the manufacturer’s protocol. qPCR was carried out using IQ™ SYBR Green Supermix (Bio-Rad, Berkeley, USA) on a Mastercycler® ep realplex (Eppendorf, Hamburg, Germany) with GAPDH as the endogenous control. Cycling conditions were: initial denaturation at 95 °C for 3 min, followed by 40 cycles of 95 °C for 15 s, 60 °C for 30 s, and 72 °C for 30 s. Relative expression levels were calculated using the 2–ΔΔCt method.

### Cell viability assay

Cells were seeded in 96‑well plates at a density of 1 × 10^3^ cells per well in 100 μL complete medium and cultured for 24 h until reaching approximately 50–60% confluence. For time‑course experiments, cells were incubated for 1 to 4 days post-treatment. At each time point, 10 μL of MTT solution (5 mg/mL in PBS, final concentration 0.5 mg/mL) was added to each well and incubated for 4 h at 37 °C. The medium was then carefully removed, and 150 μL of dimethyl sulfoxide (DMSO; Sigma) was added to each well to dissolve the formazan crystals. The plates were gently shaken for 10 min at room temperature before measuring the absorbance. For drug sensitivity assays, cells were seeded in 96‑well plates at 1 × 10^4^ cells per well in 200 μL RPMI‑1640 medium and cultured for 24 h. Cells were then treated with varying concentrations of diamminedichloroplatinum (DDP) and epirubicin (EPI) (concentrations chosen based on clinically relevant peak plasma levels) for 24 h. After treatment, 20 μL of 5% MTT was added per well, and the plates were incubated for an additional 4 h at 37 °C. Subsequently, the culture supernatant was removed, 150 μL DMSO was added, and the plates were shaken for 10 min. Absorbance was measured using an MK3 enzyme mark instrument (Bio‑Tek Instruments Inc., Winooski, VT, USA) at 490 nm. The inhibitory rate of cells was calculated as (1—mean absorption value _resistance group_/mean absorption value _control group_) × 100%, and the median inhibitory concentration (IC50) was identified.

### Colony formation assay

A 1% agar solution was prepared by dissolving agar (Sigma) in distilled water, then heated in a microwave until completely melted. The agar solution was cooled to 40 °C in a water bath. An equal volume of pre-warmed 2 × DMEM (supplemented with 20% FBS and antibiotics: 100 U/mL penicillin and 100 μg/mL streptomycin) was mixed with the melted agar and kept at 40 °C for at least 30 min to ensure homogeneity. Approximately 2 mL of this mixture was dispensed into each well of a 6-well plate and left at room temperature in a laminar flow hood for at least 1 h to allow the agar to solidify completely. Cells were then seeded on top of the agar at a final density of 2 × 10^3^ cells per well in 2 mL of DMEM containing 10% FBS. The plates were incubated at 37 °C in a humidified 5% CO₂ atmosphere, and the culture medium was replaced every 3 days. After 21 days, colonies were fixed with 100% methanol for 10 min at room temperature, stained with 2% Giemsa solution (Merck, New York, USA) for 20 min, washed with distilled water, and air-dried. Colonies consisting of more than 50 cells were counted under a light microscope. All experiments were performed in triplicate.

### Wound-healing assay and invasion assay

For the wound-healing assay, cells (1 × 10⁶ per plate) were seeded in 6-cm dishes that had been pre-coated with 10 μg/mL type I collagen (Sigma, St. Louis, MO, USA). For coating, 2 mL of the collagen solution was added to each dish, incubated at room temperature for 1 h, then aspirated and rinsed gently with sterile PBS. After seeding, cells were incubated at 37 °C in a humidified atmosphere with 5% CO₂ for 24 h until a confluent monolayer was formed. The monolayer was then disrupted by creating a straight “wound” using a sterile 200 μL pipette tip (or cell scraper). After scratching, the cells were gently washed twice with PBS to remove debris and floating cells. Phase-contrast images were captured at 0 h (immediately after scratching) and 24 h later using an Olympus CKX41 inverted microscope (Olympus, Tokyo, Japan) equipped with a digital camera. Wound closure was quantified by measuring the distance between wound edges at several predefined points using ImageJ software, and the percentage of wound closure was calculated.

Transwell inserts with an 8 μm pore size (Costar, Corning, NY, USA) were used for the invasion assay. The upper surface of the insert membrane was coated with 200 μL of Matrigel (RD, Carlsbad, CA, USA) diluted to a final concentration of 200 μg/mL in cold serum-free DMEM. The coated inserts were incubated at 37 °C for 1 h to allow the Matrigel to solidify. Next, 2 × 10^4^ cells in 200 μL serum-free DMEM were seeded into the upper chamber of each insert. The lower chamber was filled with 600 μL DMEM supplemented with 10% FBS, serving as a chemoattractant. The plate was then incubated for 24 h at 37 °C in a humidified 5% CO₂ atmosphere. After incubation, non-invading cells on the upper surface of the membrane were removed gently using a cotton swab. Cells that had invaded through the Matrigel and reached the lower surface of the membrane were fixed with cold 100% methanol for 10 min at room temperature and then stained with DAPI (Sigma; final concentration 1 μg/mL) for 5 min in the dark. Inserts were washed with PBS, and air-dried, and then the invading cells were counted in at least five random fields per insert under an Olympus BX51 microscope (Olympus, Tokyo, Japan) at 200 × magnification. Three independent inserts were used per condition, and the average number of invading cells was calculated.

### Immunofluorescence staining

Untransfected or transfected cells were grown on glass coverslips and were allowed to attach for 24 h before staining. The coverslips were washed, fixed in 3.7% formaldehyde for 15 min at room temperature, immersed sequentially in cold methanol (5 min at −20 °C) and acetone (2 min at −20 °C), and then allowed to air-dry at room temperature. After rehydration in PBS for 5 min, coverslips were blocked with 5% bovine serum albumin (BSA) in PBS for 1 h at room temperature to reduce non-specific binding. The dry coverslips were incubated with diluted primary antibodies against ADAR (1:1000; Dallas, TX, USA) and TRIM47 (1:2000; Dallas, TX, USA) overnight at 4 °C, and subsequently with Cy3- or FITC-conjugated secondary antibodies (Jackson Immuno Research Inc., West Grove, PA, USA) for 1 h at room temperature in the dark. Nuclei were counterstained with DAPI. Images were captured using a Carl Zeiss LSM 880 confocal microscope equipped with a 63x/1.4 oil immersion objective. Excitation and emission wavelengths were set as follows: DAPI (Ex: 405 nm, Em: 460 nm), FITC (Ex: 488 nm, Em: 520 nm), and Cy3 (Ex: 550 nm, Em: 570 nm). Image processing and fluorescence quantification were performed using Zeiss ZEN software (Blue Edition, v3.1).

### Western blotting

Equal amounts of protein (20–40 μg per lane) extracted from fresh tissues and cells were separated by sodium dodecyl sulfate–polyacrylamide gel electrophoresis, transferred onto nitrocellulose membranes (Millipore, Bedford, MA, USA), and subjected to immunoblot analyses. Blotting was performed with primary antibodies targeting TRIM47, ADAR, snail 1, MMP9, NCAD, ECAD, P27, PCNA, cyclin D1, RAD51, γH2AX (Santa Cruz, Dallas, TX, USA) and β-actin (Sigma, St. Louis, MO) overnight at 4 °C, followed by horseradish peroxidase-conjugated secondary antibody (1:2000, Sigma, St. Louis, MO) for 1 h at room temperature. Bands were visualized using the enhanced chemiluminescence kit (Santa Cruz, Dallas, TX, USA). Images were acquired using a ChemiDoc MP Imaging System (Bio-Rad, Berkeley, CA, USA). The densitometry of protein bands was quantified using Quantity One software (Bio-Rad).

### Immuno-histochemistry and staining evaluation

Tissue specimens were fixed in 10% PBS-buffered formalin overnight at 4 °C, embedded in paraffin, sectioned at 4 μm thickness using a microtome, and mounted onto polylysine-coated slides. After deparaffinization in xylene and rehydration through graded ethanol, antigen retrieval was performed by boiling sections in citrate buffer (pH 6.0) in an autoclave at 121 °C for 3 min. The slides were allowed to cool to room temperature before blocking endogenous peroxidase with 0.3% H₂O₂ in methanol for 15 min. Non-specific binding was blocked by incubating sections in 10% normal goat serum (Gibco) in PBS for 20 min. Primary antibody against TRIM47 (1:150, Abcam, Cambridge, UK) was applied overnight at 4 °C, while control slides received either PBS alone or non-specific purified rabbit IgG (Sigma). After washing, sections were incubated with a biotinylated secondary antibody using the ChemMate EnVision Kit (DAKO, Hamburg, Germany) for 15 min, followed by diaminobenzidine (DAB, Maixin Biotech, Fuzhou, China) for color development. Sections were counterstained with hematoxylin (Maixin Biotech), dehydrated, and mounted.

All stained sections were examined independently by two experienced pathologists in a double-blinded manner. Staining intensity (0 = negative, 1 = weak, 2 = moderate, 3 = strong) and percentage of positive tumor cells (0 = 0%, 1 = 1–30%, 2 = 31–60%, 3 = > 60%) were combined to obtain an EI (extent × intensity) score ranging from 0 to 9. ImageJ software was also used for automated quantification. Tumors were classified into two groups: low TRIM47 expression (score ≤ 3) and high TRIM47 expression (score > 3).

### Co-immunoprecipitation (Co-IP) and Mass spectrometry

Co-immunoprecipitations were performed by the Co-IP kit (Thermo Fisher Scientific, Waltham, MA). Briefly, the TRIM47 cDNA was inserted into the Flag-tagged vector, with *Nhe*I and *Sal*I sequences of these two restriction enzymes at both terminals inserted into the His-tagged vector to form two recombinant plasmids. The recombinant plasmid carrying the target gene and the virus packaging plasmid were co-transfected into THJ-29 T cells. The corresponding stable transfected cells were screened in the culture system. The His-tagged overexpression stable transfected cells and TRIM47 overexpression stable transfected cells were obtained. After the extraction of the immune-precipitated protein, the antibody was combined with protein A/G agarose, and then the bound antibody was crosslinked, followed by pre-clearing of the protein sample, antigen immunoprecipitation, and antigen elution before Western Blot detection was performed.

Mass spectrometry analysis was conducted by Applied Protein Technology (Shanghai, China) using 5800 MALDI-TOF/TOF (AB Sciex, USA). Before analysis, protein samples were prepared by enzymatic digestion with trypsin, resulting in peptide mixtures suitable for mass spectrometric analysis. The resulting peptides were mixed with a matrix solution and applied to a MALDI target plate. The matrix-assisted laser desorption/ionization (MALDI) process facilitated the ionization of peptides, which were then analyzed using the mass spectrometer. For quantitative analysis, proteins were considered differentially expressed if they exhibited a minimum of one unique peptide match and a fold change of ≥ 3.0 or ≤ 3.0, with a p-value < 0.05. Protein identification was achieved by matching observed peptide masses to a protein database, ensuring accurate and reproducible results.

### Xenograft assay *in vivo*

All animal procedures were approved by the Institutional Animal Care and Use Committee. Male BALB/c-nu mice (4–5 weeks old, weighing 18–20 g) were obtained from Shanghai Laboratory Animal Company (SLAC, Shanghai). Mice were acclimatized for 1 week in a pathogen-free environment with controlled temperature (22 ± 2 °C) and a 12-h light/dark cycle, with ad libitum access to food and water. Cells were harvested during the logarithmic phase and resuspended in serum-free RPMI-1640 medium at a concentration of 1 × 10⁷ cells/mL. Mice were randomized into two groups (n = 5 per group): the control group and the TRIM47-sh#1 group. Each mouse was anesthetized briefly with isoflurane and then injected subcutaneously in the upper right thigh with 0.2 mL of the cell suspension. Tumor growth was monitored every 3 days using calipers. Mice were observed for a total period of 3 weeks. At the end of the study, mice were euthanized by CO₂ asphyxiation, and tumors were excised, weighed, and processed for further analysis.

### Statistical analysis

Data are presented as mean ± standard deviation (SD) and are derived from three independent experiments. Before statistical analysis, the normality of the data was assessed using the Shapiro–Wilk test. For data that met normal distribution, the independent sample t-test was used to compare the differences between the two groups; for data that did not meet normal distribution, the Mann–Whitney U test was used. When three or more groups were compared, a one-way analysis of variance (ANOVA) was used. In the case of significant ANOVA results, Tukey's HSD (Honestly Significant Difference) post hoc test was used for multiple comparisons to determine which groups were significantly different. All statistical analyses were performed using the SPSS 13.0 software package (SPSS Inc., IL, USA). P values ​​were two-sided tests, and P < 0.05 was considered statistically significant. Assuming a two-tailed test with an alpha level of 0.05 and a desired power (1–β) of 0.8, the sample size was calculated using the formula for comparing two independent means: $n=\frac{{\left({Z}_{1-a/2}+{Z}_{1-\beta }\right)}^{2}\times {2\sigma }^{2}}{{\delta }^{2}}$ where Z1 − α/2 is approximately 1.96 and Z1 − β is approximately 0.84. Our calculations indicated that a sample size of approximately 20–25 per group was necessary. Accordingly, we included 24 samples in each group, which is sufficient to achieve statistical power and robust conclusions. Similarly, for in vivo experiments, sample sizes were determined based on previous studies in similar experimental settings, which demonstrated that using 5 mice per group provides adequate power to detect significant differences in tumor growth and other outcomes. In vitro experiments were performed in triplicate and repeated independently to ensure reproducibility and statistical validity.

## Results

### Increasing expression of TRIM47 was associated with the clinic-pathological features of thyroid carcinoma

**Fig. 1:**
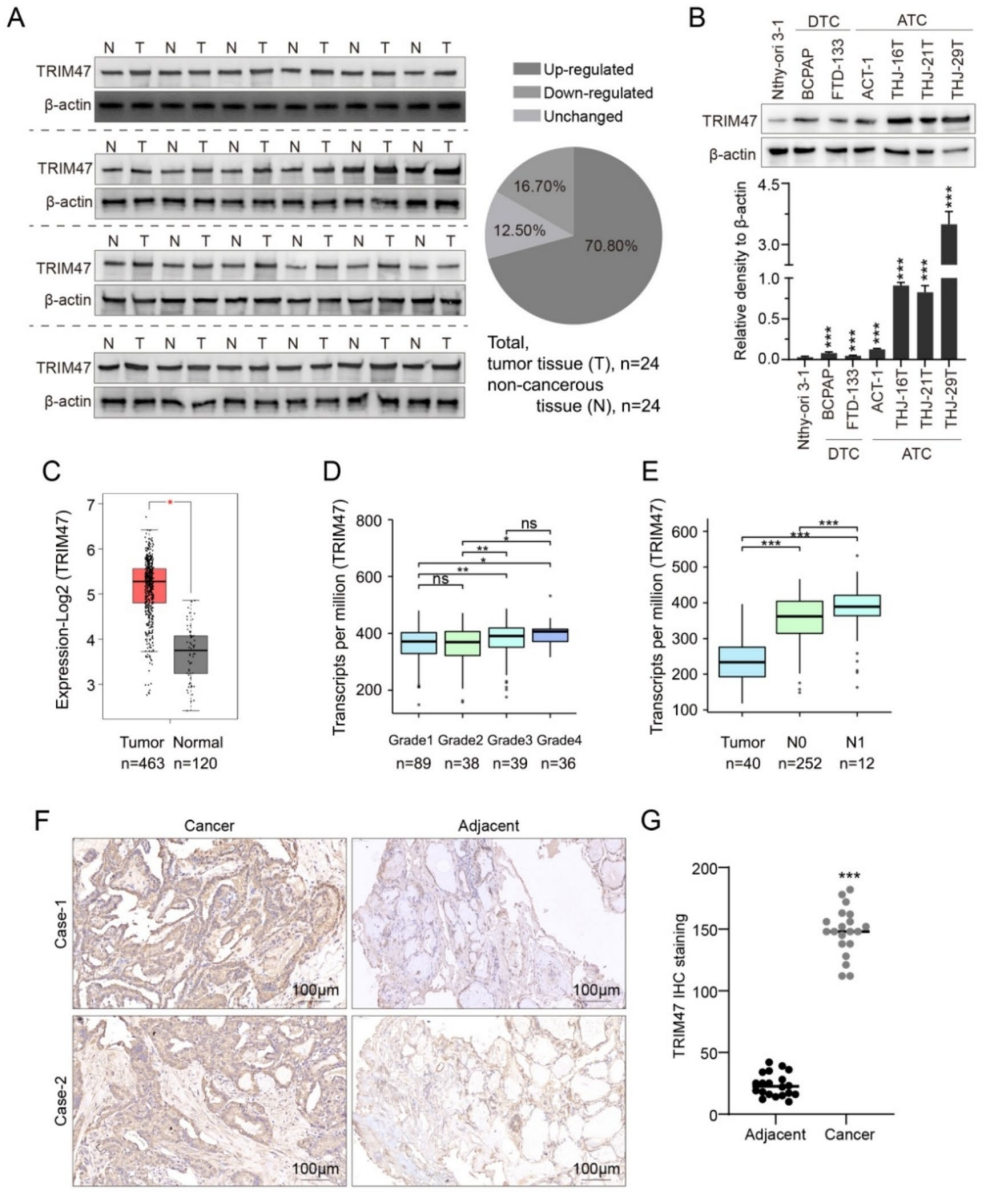
TRIM47 was highly expressed in human thyroid cancer, especially in ATC. **A** The expression of TRIM47 protein was detected in tumor (T) and normal(N) tissues (left), and 70.8% of the samples were elevated in T group (*n* = 24 for each group). **B** The protein levels of TRIM47 in DTC cells and ATC cells were detected by Western Blotting analysis in differentiated thyroid carcinoma (DTC) cell lines including BCPAP and FTD-133, and anaplastic thyroid carcinoma (ATC) cell lines including ATC-1, THJ-16 T, THJ-21 T and THJ-29 T compared to human normal thyroid cell line (Nthy-ori 3–1) (*n* = 3). **C** GEPIA database showed the difference of TRIM47 expression between cancer and adjacent cancer. **D** Anslysis based on TCGA database showed that T3-4 stages expression was significantly higher than T1-2 stages. **E** TCGA database showed that TRIM47 expression level was closely related to N stage. **F**, **G** IHC detected the expression of TRIM47 in the samples (*n* = 3 for panel F). Scar bar = 100 μm * *p* < 0.05, ** *p* < 0.01, *** *p* < 0.001 vs. normal thyroid cell line

We inspected TRIM47 expression in clinical TC (T; *n* = 24) and non-cancerous (N; *n* = 24) tissues. As shown in Fig. 1A, 70.8% of the samples increased in protein levels of TRIM47 in tissues of thyroid carcinoma than in non-cancerous tissues. Western Blotting was used to detect the protein level of TRIM47 in human normal thyroid cell line (Nthy-ori 3–1), differentiated thyroid carcinoma (DTC) cell lines (BCPAP, FTD-133), and anaplastic thyroid carcinoma (ATC) cell lines (ATC-1, THJ-16 T, THJ-21 T and THJ-29 T). The TRIM47 protein expression was markedly higher in DTC cell lines than in the Nthy-ori 3–1 cell lines (Fig. 1B, all *p* < 0.001). Especially, the protein level of TRIM47 in ATC was dramatically higher than that in Nthy-ori 3–1 (Fig. 1B, all *p* < 0.001). In comparison between DTC and ATC cells, the TRIM47 showed extra-significant higher levels in ATC than in DTC cells (Fig. 1B, all *p* < 0.001).

Our analysis of the databases was consistent with the above results. GEPIA database showed that the expression of TRIM47 in the cancer tissues was much higher than that in adjacent tissues (Fig. 1C). TCGA database showed that the expression of TRIM47 in grades 3 and 4 was significantly higher than that in grades 1 and 2 (Fig. 1D). Furthermore, the expression of TRIM47 was closely related to the metastasis stage (N0 and N1), in which the higher expression of TRIM47along with the higher N0 (no regional lymph node metastasis) and N1 (metastases in 1 to 3 axillary lymph nodes) stages with all *p* < 0.01 (Fig. 1E). The IHC inspection verified the above analysis shown in Fig. 1F-G.TRIM47 was detected in pathological tumor tissues in the most pericellular distribution of basement cell layers of the disordered thyroid gland with strongly increased expression (Fig. 1F left and G) than that in their adjacent tissues (Fig, 1F right and G). Thus, we would like to claim that the alternation of the expression of TRIM47 relates to clinicopathological features of thyroid carcinoma.

### TRIM47 promoted the growth of thyroid cancer cells and TRIM47 knocking-down enhanced the chemo-sensitivity of thyroid cancer cells

To further evaluate the association of TRIM47 with TC malignancies, we analyzed TRIM47 protein levels in two TC cell lines (THJ-29 T and FTD-133). Short hairpin RNAs (shRNA) specific against TRIM47 were constructed into lentiviral vectors specific against the gene used to knock down endogenous TRIM47 in THJ-29 T cells. As the shRNA constructs that made highly efficient inhibition of TRIM47 expression, which we named Sh-TRIM47-#1 (short for Sh1), Sh-TRIM47-#2 (short for Sh2), and Sh-TRIM47-#3 (short for Sh3), we employed then applying in the following tests. Construction of TRIM47expressing plasmids was achieved for overexpression of TRIM47 in FTD-133 cell line with the vector alone without the inserts as control.

**Fig. 2:**
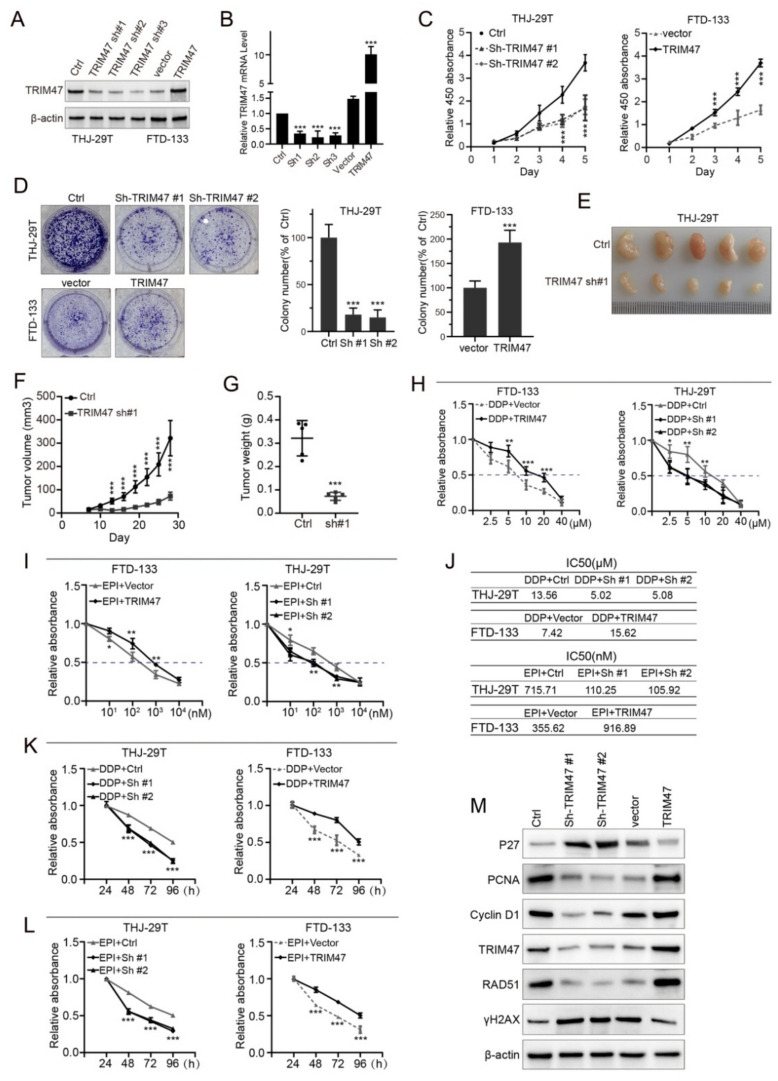
TRIM47 promoted the growth of thyroid cancer cells and TRIM47 knocking-down enhanced the chemo-sensitivity of thyroid cancer cells. **A** The expression of TRIM47 protein were detected after transfected with TRIM47 sh#1, TRIM47 sh#1 and TRIM47 sh#1 specific against TRIM47 and detected with Western Blotting analysis (*n* = 3). **B** The expression of TRIM47 mRNA were detected with quantitative PCR on cells transfected with TRIM47 sh#1, TRIM47 sh#1 and TRIM47 sh#1 specific against TRIM47 compared to three controls including non-transfection, and vector alone and TRIM47 cDNA (*n* = 3). **C**, **D** Both CCK8 array and colony growth array were applied to detect the regulation of TRIM47 on cell proliferation in THJ-29 T and FTD-133 cell lines (*n* = 3). **E**–**G** Subcutaneous tumorigenesis in nude mice after TRIM47 knocking-down in THJ-29 T, with tumor growth curve and tumor weight statistics (*n* = 5). **H**, **J** Determination of DDP EPI resistance concentration in a range of 10 to 10 × 10^3^ nM of EPI (**I**) and 2.5 to 40 μM of EPI (H) in THJ-29 T and FTD-133 based on the grouping (*n* = 3). **K**, **L** Determination of cell viability via treatments of DDP with knocking-down and overexpression of TRIM47 in timing spectrum of 24–96 h in THJ-29 T and FTD-133 (**K**), and via treatments of DDP and EPI in timing spectrum of 24–96 h in THJ-29 T and FTD-133 (L) (*n* = 3). M: Western Blotting analysis on protein markers related to proliferation and DNA damage including P27, PCDA, cyclin D1, RAD51 and γH2AX 1 along with TRIM47 and internal control β-actin within knocking-down and overexpression of TRIM47 cells (*n* = 3). * *p* < 0.05, ** *p* < 0.01, *** *p* < 0.001 vs normal

Western Blotting analysis and quantitative PCR verified that both inspecting systems were successfully constructed (Fig. 2A-B). Both abilities of the CCK-8 and in vitro colony formation were decreased in response to TRIM47 silencing in THJ-29 T, and were increased in response to TRIM47overexpressingwhile treatments of Sh-TRIM47-#1 and Sh-TRIM47-#2 in culture THJ-29 T and FTD-133 (Fig. 2C). The significance of the TRIM47 silencing in THJ-29 T initiated at the culture day 4 (*p* < 0.001) and 5 (*p* < 0.001) is shown in Fig. 2C left. After TRIM47 was transfected in FTD-133, the overexpression began to generate significant exogenous TRIM47 on the third day of transfection and lasted 3 three days at least (all *p* < 0.001). In colony growth inspection, the RNAi via Sh-TRIM47-1 and Sh-TRIM47-2 specific against TRIM47 significantly limited the proliferation of THJ-29 T (Fig. 2D top left and middle; *p* < 0.001). While both Sh-TRIM47-1 and Sh-TRIM47-2 exhibited high efficiency against THJ-29 T colony formation, the overexpression of TRIM47 promoted FTD-133 colony growth (Fig. 2D bottom left and right* p* < 0.001).

Our data on subcutaneous tumors induced with inoculation of THJ-29 T cells in nude mice showed that the tumor grew significantly slower and smaller in response to TRIM47 silencing (Fig. 2E-G). 30-day after cells xenografted into nude mice, Sh-TRIM47-#1 treated cells produced much smaller tumors than non-treated cells with distinguishing significance initiated from the 13th to 27th days (Fig. 2F), and the final weights were dramatically different (Fig. 2G; *p* < 0.001).

Furthermore, we examined the drug resistance effect on the concentration of cisplatin or cis-DDP II and EPI. We approved that the half maximal inhibitory concentration (IC50) of DDP was decreased in response to TRIM47 silencing in DTD-133 (Fig. 2H left and J), and was increased in response to TRIM47 overexpressing in THJ-29 T (Fig. 2H right and J), with DDP spectrum of 2.5, 5, 10, 20 and 40µM. We also ratified that the half maximal inhibitory concentration (IC50) of EPI was decreased in response to TRIM47 silencing in DTD-133 (Fig. 2I left and J), and were increased in response to TRIM47 overexpressing in THJ-29 T (Fig. 2I right and J), with EPI spectrum of 10, 10^2^, 10^3^ and 10^4^ nM.

The cell viability with the treatment of DDP and EPI showed that the viability was decreased in response to TRIM47 silencing in THJ-29 T, and was increased in response to TRIM47 overexpressing (Fig. 2K and L). While TRIM47 silencing with both Sh-TRIM47-#1 and Sh-TRIM47-#2, DDP caused the decreased cell viability began at 48 h (*p* < 0.001) via 48 h (*p* < 0.001) and lasted till 96 h (Fig. 2K left). While TRIM47 expression, DDP caused the increased cell viability began at 48 h (*p* < 0.001) via 48 h (*p* < 0.001) and lasted till 96 h (Fig. 2K right) correspondingly. Similarly, with the treatment of EPI, TRIM47 silencing with both Sh-TRIM47-#1 and Sh-TRIM47-#2, EPI caused the decreased viability initiated at 48 h (*p* < 0.001) via 48 h (*p* < 0.001) and lasted till 96 h (Fig. 2L left). While TRIM47 expression, EPI caused the increased cell viability began at 48 h (*p* < 0.001) via 48 h (*p* < 0.001) and lasted till 96 h (Fig. 2K right) as well.

Based on our experiments above, the expression of TRIM47 was closely related to TC proliferation and enhanced chemo-sensitivity, so we further inspected the critical genes associated with proliferation and DNA damage. Comprehensively, our findings unveiled that TRIM47 exhaustion dramatically down-regulated PCNA, cyclin, and RAD51 expression, and significantly raised the expression of P27 and γH2AX while silencing TRIM47 with Sh-TRIM47-1 and Sh-TRIM47-2 (Fig. 2M). Meanwhile, the TRIM47 overexpression significantly up-regulated the expressions of PCNA, cyclin D1, and RAD51 along with the down-regulation of P27 and γH2AX (Fig. 2M). According to our experiments and analysis above, we claimed that TRIM47 promoted the growth of thyroid cancer cells, and deleting expression by *TRIM47* knocking-down enhanced the chemo-sensitivity of thyroid cancer cells.

### TRIM47 silencing suppressed the migration and invasion of thyroid carcinoma cells *in vitro*

Migration and invasion of tumor cells through their cellar membrane have been a significant procedure in the cascade of metastasis and then we used this membrane to inspect the possible function of TRIM47on migration and invasion in cell culture system using differentiated thyroid carcinoma cell line FTD-133 and anaplastic thyroid carcinoma cell line THJ-29 T with classic approaches including wound-healing and Matrigel invasion assays as long with in vivo xenograft assay.

**Fig. 3:**
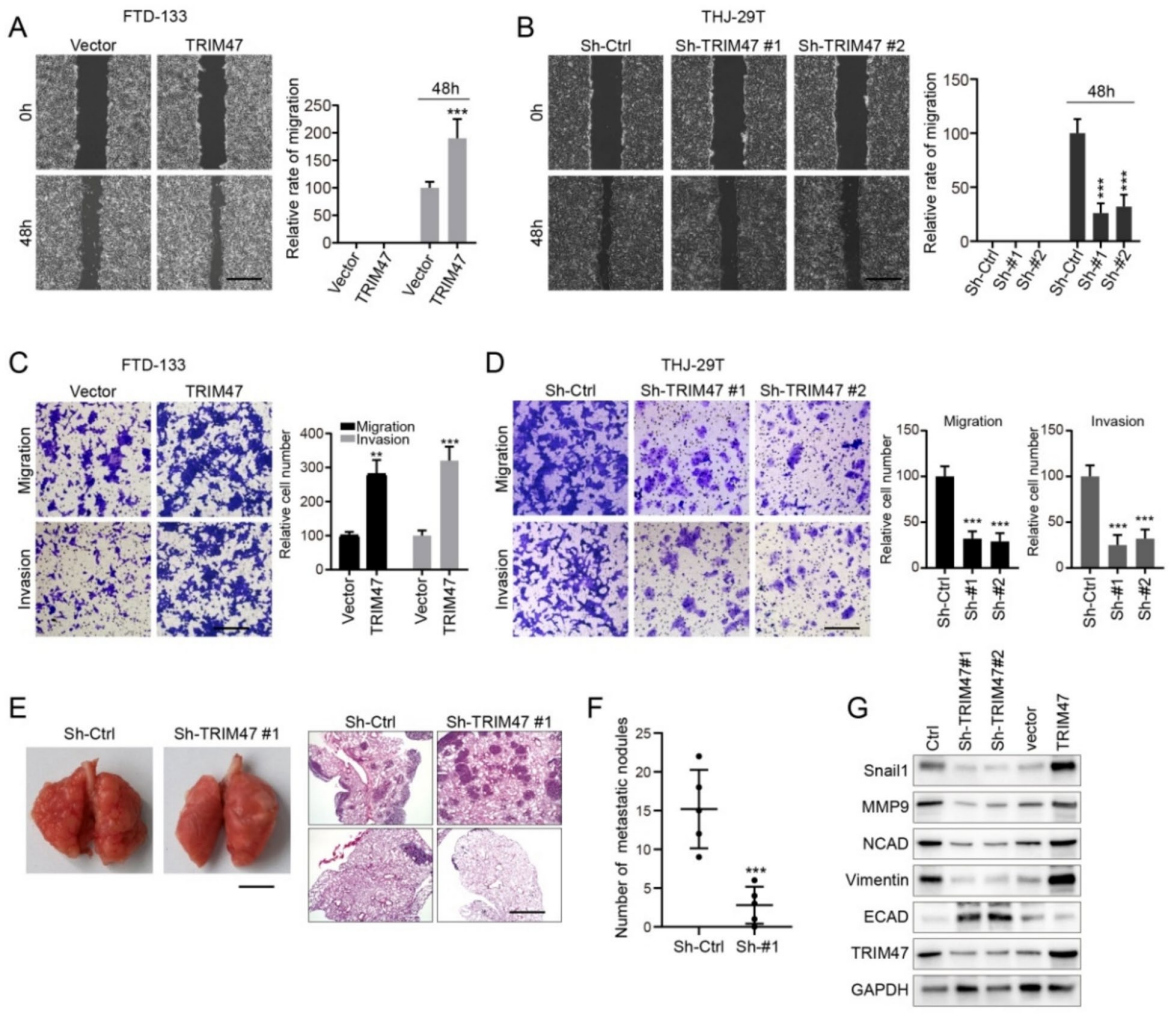
TRIM47 regulated the migration and invasion of thyroid cancer cells. **A**, **B** Wound-healing assay showed that the mobility of THJ-29 T and FTD-133 cells was evidently reduced after TRIM47 overexpressed (**A**) and TRIM47 knocking-down with TRIM47 sh#1, TRIM47 sh#1 and TRIM47 sh#1 specific against TRIM47 (**B**) compared to vector sh# control in FTD-133 and THJ-29 T cells (*n* = 3). C-D: TRIM47 overexpression significantly increased migration and invasion of THJ-29 T and FTD-133 cells in matrigel invasion assays (**C**), and TRIM47 knocking-down significantly lessen migration and invasion of THJ-29 T and FTD-133 cells in matrigel invasion assays (**D**) (*n* = 3). E–F: TRIM47 silencing, the tumor in nude mice was smaller than that in sh-control and the obsessive consequences in sh-control were better with fewer of metastatic nodules in lung (*n* = 5). G: Western Blotting analysis inspected the cell migration related markers including Snail 1, MMP9, NCAD, Vimentin, ECAD along with TRIM47 and internal control GAPDH (n = 3). * *p* < 0.05, ** *p* < 0.01, *** *p* < 0.001 vs normal

To examine whether TRIM47 acted a role in controlling the migration and invasion of ATC THJ-29 T and DTC FTD-133 cells, the wound-healing assay was applied and showed that the mobility of THJ-29 T and FTD-133 cells was increased with the over-expression of TRIM47 cDNA in 48 h compared to 0 h with (Fig. 3A;* p* < 0.001). Oppositely, while silencing TRIM47 with Sh-TRIM47-1 and Sh-TRIM47-2, the mobility of THJ-29 T and FTD-133 cells reduced in 48 h compared to 0 h within both Sh-TRIM47-#1 (*p* < 0.001) and Sh-TRIM47-#2 (*p* < 0.001) showed in Fig. 3B. Furthermore, when TRIM47 was over-expressed in DTC FTD-133 cells, the mobility migration and invasion cells were increased in both categories of migration (*p* < 0.01) and invasion (*p* < 0.001) of FTD-133 shown in Fig. 3C. While knocking of TRIM47 significantly in 48 h, the migration and invasion of THJ-29 T cells in Matrigel invasion assays were significantly lessened on both categories of migration (*p* < 0.01) and invasion (*p* < 0.01) as well exhibited in Fig. 3D.

The molecular mechanism of TRIM47 involving lung metastasis was next carried out in vivo xenograft assay with anaplastic thyroid carcinoma THJ-29 T cells treatedTRIM47 silencing with Sh-TRIM47-#1. Upon the TRIM47 silencing, the tumor was smaller and the obsessive consequences were better with fewer metastatic nodules in the lung after 28 days of the cells xenografted (Fig. 3E and F). Furthermore, we examined the cell migration marker E-cadherin (ECAD) and the characteristic pattern of the protein expression pattern of mesenchymal cells including Snail 1, MMP9, NCAD, and vimentin. While TRIM47 silencing with Sh-TRIM47-#1 and Sh-TRIM47-#2, we found that ECAD was significantly up-regulated and Snail 1, MMP9, NCAD, and vimentin were down-regulated. Upon TRIM47 over-expression, ECAD was significantly down-regulated and Snail 1, MMP9, NCAD, and vimentin were up-regulated (Fig. 3G). E-cadherin upregulation along with the expression of Snail 1, MMP9, NCAD, and vimentin, which was characteristic of mesenchymal cells, being significantly suppressed after TRIM47 reduced expression suggests that the TRIM47 silencing worsened the cell migration and invasion of thyroid carcinoma by ablating epithelial-mesenchymal transition (EMT).

### TRIM47 promoted thyroid tumorigenesis *via* down-regulation of ADAR

**Fig. 4:**
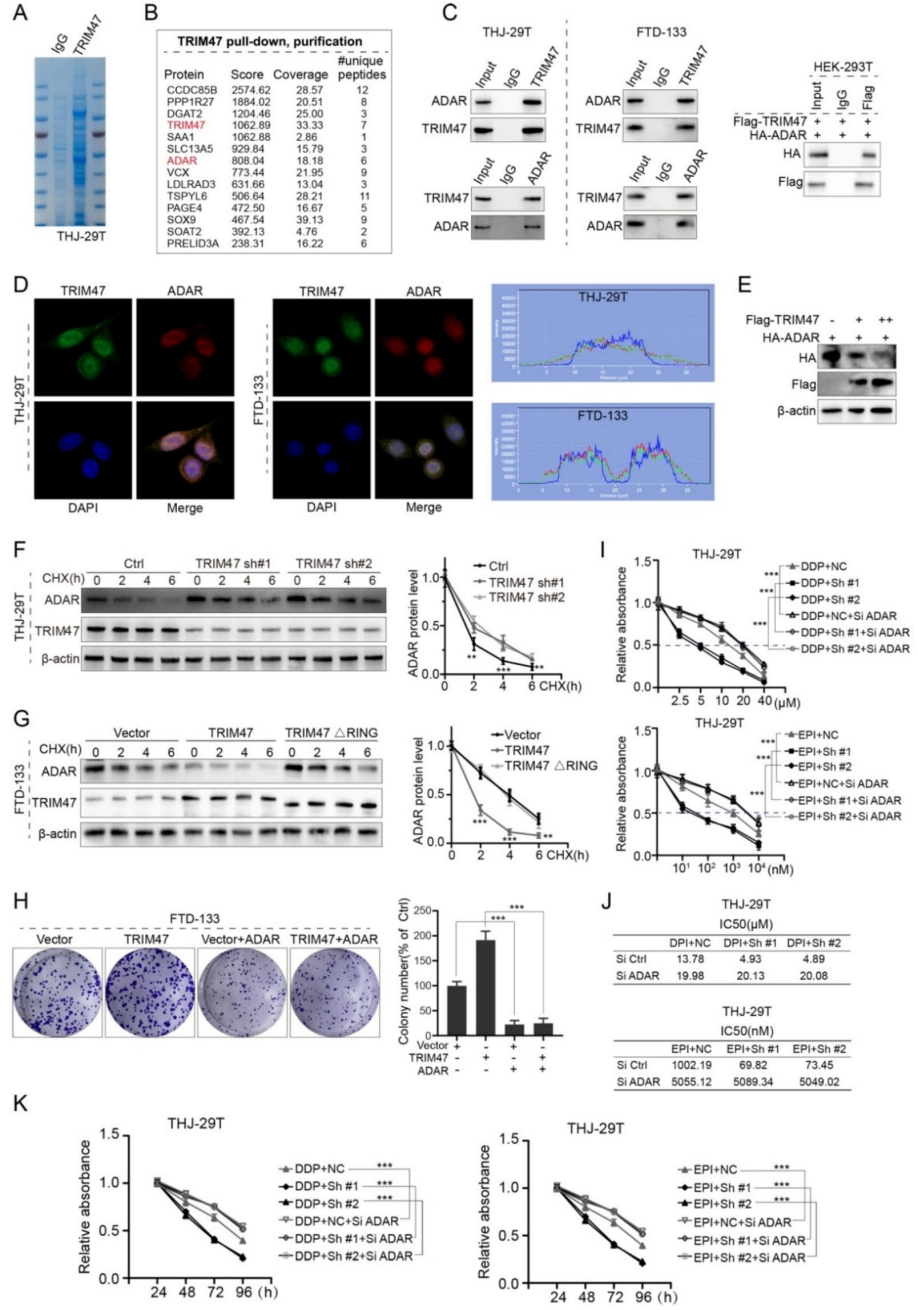
TRIM47 along with ADAR controlled thyroid tumorigenesis. **A**, **B** Co-IP protein mass spectrometry of TRIM47 complex precipitated from differentiated thyroid carcinoma THJ-29 T cells. **C** The interaction between endogenous and exogenous TRIM47 and ADAR was verified in THJ-29 T and FTD-133 cell lines along with human embryonic kidney HEK293T (*n* = 3). **D** Immunofluorescence co-localization showed that TRIM47 and ADAR were highly overlapped in the cytoplasmic distribution of cultured THJ-29 T and FTD-133 (*n* = 3). **E** TRIM47 proved to have gradient-dependent concentration-ascending regulation effect on concentration-descending of ADAR at the protein level (*n* = 3). **F**, **G** Knocking-down and overexpression of TRIM47 to verify the regulation of ADAR protein stability in THJ-29 T and FTD-133 as TRIM47 ring is an inactive type of TRIM47 enzyme. Colony formation approach supported the regulation relationship of TRIM47 and ADAR (*n* = 3). H–K: ADAR reduced the promoting effect of TRIM47 on cell proliferation (**H**), sensitization effect of chemotherapeutic drugs (**I**), and controlling capability on cell viability (**K**) (*n* = 3). * *p* < 0.05, ** *p* < 0.01, *** *p* < 0.001 vs control

To comprehend the cellular mechanism of TRIM47 in thyroid tumorigenesis, we carried out mass spectrometry with the precipitation of immunoprecipitation (IP) TRIM47 complex and further made sure of their transcription level of components of the complex with mass spectrometry. Our IP protein mass spectrometry of TRIM47complex indicated a tight relationship with ADAR (Fig. 4A) indicated with Red in Fig. 4B. Further, we examined the expression of ADAR and TRIM47 in differentiated thyroid carcinoma FTD-133 cell line and anaplastic thyroid carcinoma THJ-29 T cell line. The ADAR and TRIM47 associated individual pull-downs blotted and analyzed with antibodies specific against TRIM47 and ADAR showed that TRIM47 and ADAR interacted with each other in the cultured ATC THJ-29 T and DTC FTD-133 cells (Fig. 4C left and middle). Furthermore, we overexpressed HA-ADAR and Flag-TRIM47 in human embryonic kidney HEK-295 T cells and also approved the interaction between ADAR and TRIM47 in non-thyroid carcinoma cell lines (Fig. 4C right).

Using the culture cells combined with immunofluorescence analysis, we applied fluorescence co-localization to identify the cytoplasmic distribution of TRIM47 and ADAR proteins DTC FTD-133 and ATC THJ-29 T cells. Our data showed that TRIM47 and ADAR were highly overlapped in the distribution of subcellular structures, which is most likely co-localized in the endoplasmic reticulum (ER) and nuclei of both THJ-29 T (Fig. 4D left and right) and FTD-133 (Fig. 4D middle and right) cells. Our experiments also showed that TRIM47 has a gradient regulation effect on ADAR expression at their protein level (Fig. 4E).

Comprehensively, we approved that the regulation of TRIM47 on the stability of ADAR protein was CHX timing associated via TRIM47 overexpression and TRIM47 knocking-down with TRIM47-#1 and Sh-TRIM47-#2 (Fig. 4F-G). Within the spectrum of 0, 2, 4, and 6 h of CHX treatment, TRIM47-#1 and Sh-TRIM47-#2 generated TRIM47 knocking-down promoting the significant increase of ADAR protein (Fig. 4F left). This timing-dependent effect started at 2 h (*p* < 0.01) after CHX treatment and was maintained via 4 h (*p* < 0.001) up to 6 h (*p* < 0.01) showed in Fig. 4F right. It is critical that the ADAR down-regulation via TRIM47 overexpression was truncated TRIM47ΔRING (an inactive type of TRIM47 enzyme) dependent (Fig. 4G left), which was initiated at 2 h (*p* < 0.001) via 4 (*p* < 0.001) up to 6 h (*p* < 0.01) of CHX treatment (Fig. 4G right). In DTC FTD-133 cells, the proliferation showed with colony formation approved that the TRIM47 overexpression could directly promote the FTD-133 proliferation but was able to be terminated by ADAR over-expression in the TRIM47 plus ADAR group (Fig. 4H).

Furthermore, our data also showed that ADAR could reduce the promoting effect of TRIM47 on cell sensitization effect on chemotherapeutic drugs (Fig. 4I-J), and control cell viability as well (Fig. 4K). Based on the analysis of our data above, we would like to claim that TRIM47 promotes thyroid tumorigenesis via the downregulation of ADAR, which is consistence with both the thyroid carcinoma tissues and the cultured TC cells.

### TRIM47 impaired ADAR stability and promotes ADAR ubiquitination

**Fig. 5:**
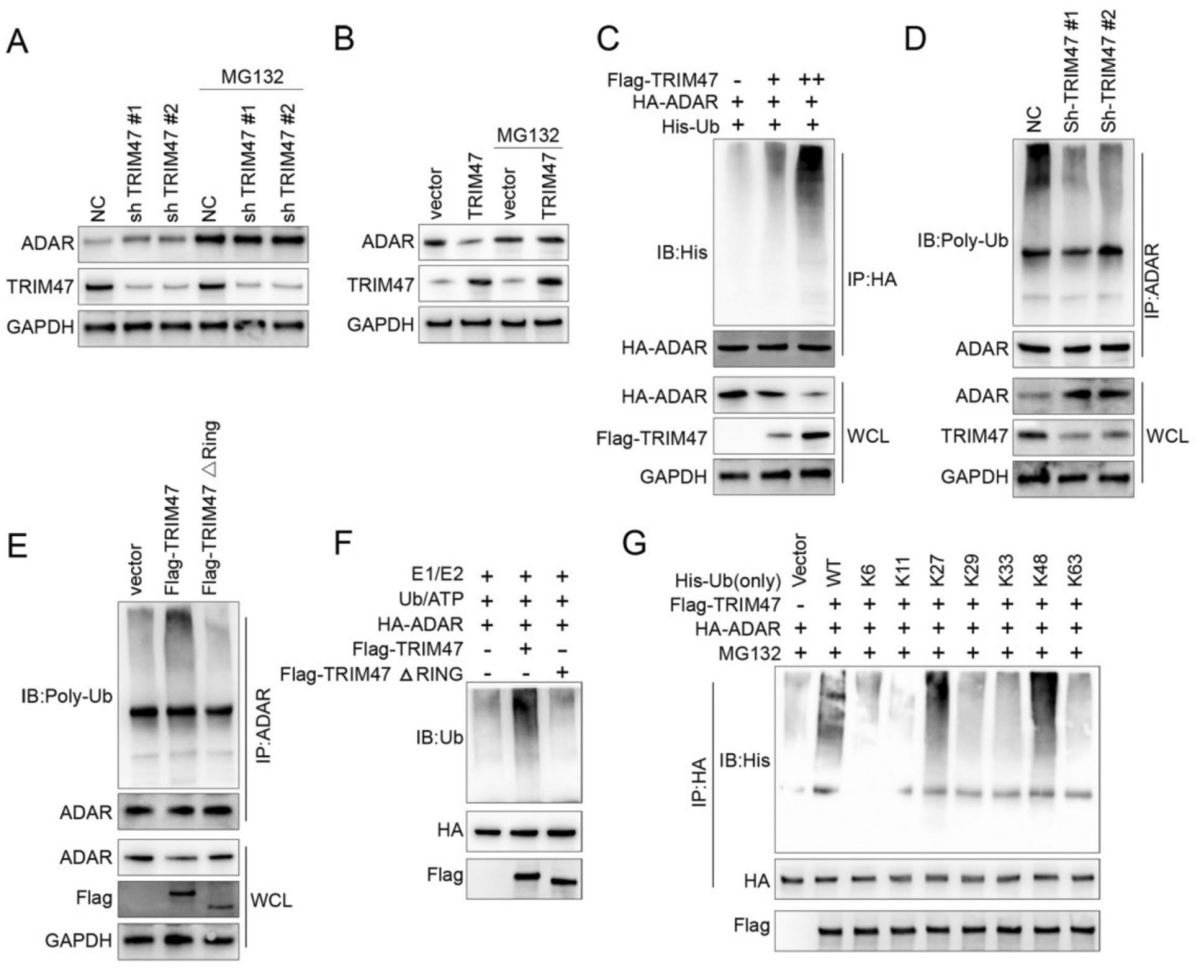
TRIM47 impairs ADAR stability and promoted ADAR ubiquitination. **A**, **B** A proteasome inhibitor MG132 rescued the regulation of ADAR in TRIM47 knocking-down and overexpression groups via Western Blotting analysis (*n* = 3). **C** Co-immunoprecipitation showed that the ubiquitination modification of ADAR promoted by TRIM47 was dose-dependent (*n* = 3). **D**, **E** TRIM47 Knocking-down regulated the ubiquitination level of ADAR which was depended on TRIM47 enzyme activity (**D**). And TRIM47 overexpression regulated the ubiquitination level of ADAR which was depended on TRIM47 enzyme activity (**E**) (*n* = 3). **F** TRIM47 promoted ADAR ubiquitination was depended on TRIM47 enzyme activity (*n* = 3). **G** The modification of TRIM47 promoted ADAR ubiquitination is mainly ubiquitin K27 and K48 molecular linkage mode (*n* = 3). * *p* < 0.05, ** *p* < 0.01, *** *p* < 0.001 vs control

To further investigate the detailed information between TRIM47 and ADAR, we applied treatment with a proteasome inhibitor MG132 to differentiated thyroid carcinoma FTD-133 cells combining with the short hairpin RNAs with TRIM47-#1 and Sh-TRIM47-#2 specific against *TRIM47*, and the *TRIM47* overexpression. As a proteasome inhibitor, our data showed that MG132 was able to rescue the regulation phenotype of ADAR by TRIM47 knocking-down (Fig. 5A) and overexpression (Fig. 5B) groups inspected with Western Blotting (Fig. 5). Immunoprecipitation IP suggested that the ubiquitination modification of ADAR by TRIM47 was dose-dependent (Fig. 5C). In vivo ubiquitination experiments showed that TRIM47 knocking-down and TRIM47 overexpression could regulate the ADAR-associated ubiquitination level (Fig. 5D). This phenomenon depended on TRIM47 enzyme activity transfected with the inactive type of TRIM47 cDNA i.e. truncated TRIM47ΔRING (Fig. 5E). In vitro*,* ubiquitination experiments showed that TRIM47 was able to promote ADAR ubiquitination, in which the phenomenon was also dependent on its enzyme activity as shown in Fig. 5F with TRIM47ΔRING.

Comprehensively, the data approved that the molecular connection mode of ubiquitination modification of ADAR by TRIM47was mainly K27 and K48but, but not K6, K11, K29, K33 and K63 via immunoprecipitation (Fig. 5G). Collectively, these results suggested that TRIM47 impairs ADAR stability and triggers the ubiquitination of ADAR within in vitro cultured FTD-133 cells.

### Phosphorylation was required for ADAR ubiquitination and stability

Because Glycogen synthase kinase 3β (GSK-3β) regulates epithelial-mesenchymal transition and cancer stem cell properties in triple-negative breast cancer (Jin et al. 2019) and we also found that TRIM47 reduced expression significantly suppressed epithelial-mesenchymal transition marketed with expression abnormality of Snail 1, MMP9, NCAD and vimentin thyroid carcinoma (Fig. 3G), we further investigated this possible interaction between TRIM47 and GSK-3β.

**Fig. 6:**
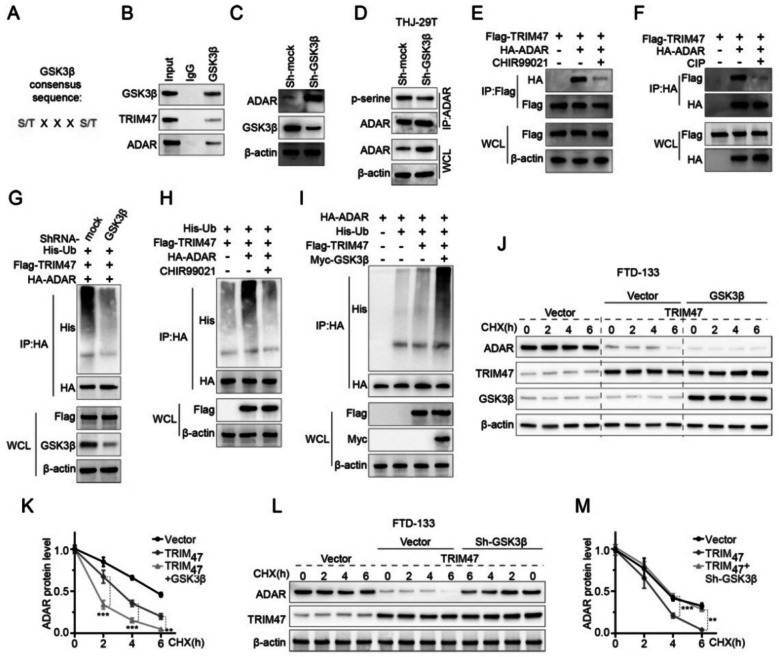
Phosphorylation is required for ADAR ubiquitination and stability. **A** Sequence characteristics of GSK3β binding protein (*n* = 3). **B** GSK3β combined with TRIM47 and ADAR at the same time inspected with Co-immunoprecipitation (*n* = 3). **C** Knocking-down GSK-3β increased the abundance of ADAR expression (*n* = 3). **D** GSK-3β Knocking-down reduced the phosphorylation of ADAR indicated with *p*-serine (*n* = 3). **E**, **F** GSK3β inhibitor CHIR99021 and phosphorylation inhibitor Myc-GSK-3β weakened the interaction between TRIM47 and ADAR (*n* = 3). **G**-**H** GSK-3β knocking-down and inhibited deceased the ADAR associated ubiquitination (*n* = 3). **I** Overexpressing GSK-3β promoted ADAR associated ubiquitination (*n* = 3). **J**-**K** Overexpression of GSK-3β promoted the ubiquitinated modification of ADAR and further weakened the stability of ADAR (*n* = 3). **L**-**M** GSK-3β Knocking-down rescued TRIM47 ability further to regulate the stability of ADAR (*n* = 3). * *p* < 0.05, ** *p* < 0.01, *** *p* < 0.001 vs control

Systematically, we further investigated the characteristic of GSK-3β binding protein with consensus sequence (Fig. 6A) and approved with immunoprecipitation that GSK-3β could combined with TRIM47 and ADAR at the same time (Fig. 6B) which was also reflected in the Co-IP mass spectrometry results of TRIM47. While GSK-3β was knocked down with the short hairpin RNAs (shRNA) sh-GSK-3β specific against GSK-3β in anaplastic thyroid carcinoma THJ-29 T cells, it triggered the increase of expression abundance of ADAR and the decrease of the hyperphosphorylation of ADAR compared to sh-mock via immunoprecipitation IP (Fig. 6C and D). When GSK-3β inhibitor (CHIR99021) and phosphorylation inhibitor (Myc-GSK-3β) were applied, the ability of TRIM47 to bind to ADAR was weakened with IP detection (Fig. 6E and F). Moreover, while GSK-3β knocking-down or GSK-3β inhibitors were carried out, the modification of ubiquitination of ADAR was inhibited (Fig. 6G-I).

Contrastingly, GSK-3β overexpression was able to promote ADAR ubiquitination and further weaken the stability of ADAR in differentiated thyroid carcinoma FTD-133 cells (Fig. 6J-K). To a certain extent, GSK-3β knocking-down was able to rescue the ability of TRIM47 expression, and further regulate the stability of ADAR in DTC FTD-133 cells (Fig. 6L). This GSK-3β mediated phosphorylation was timing dependent, which could respond to the decrease of ADAR level initiated at 4 h (*p* < 0.001) after treatment of CHX plus TRIM47 expression and Sh-GSK-3β, which would last up to 6 h (*p* < 0.01) at least (Fig. 6M). Based on the above data, we would like to claim that the phosphorylation on either TRIM47 or ADAR was required for GSK-3β associated ubiquitination and stability.

## Discussion

### Main interpretation

Members of the TRIM protein family are known to be involved in many biological procedures (Chen et al. 2021), and changes in abundance or action are associated with a variety of obsessive conditions, containing viral infections, developmental and neuron degenerative illness along cancer incidence (Li Z et al., 2021). Recent studies show that different TRIM members could both positively and negatively regulate carcinogenesis (Hatakeyama 2011). In this study, we demonstrated that TRIM47 suppression was e detected in thyroid carcinoma, especially anaplastic thyroid carcinoma. Our detailed experiments depict a novel mechanism which is TRIM47 promoted the development of thyroid carcinoma via ADAR-associated ubiquitination, and GSK-3β mediated phosphorylation on both/either TRIM47 and ADAR was critical for TRIM47 suppression.

As informed in earlier studies, TRIM47 upregulation was related to worse prognosis and promoted malignancy in several tumors including gastric cancer (Xia et al. 2021), pancreatic cancer (Li et al. 2021), breast cancer (Azuma K et al., 2021), and many other carcinomas (Azuma K 2022; Mohammadi et al. 2022). As for the actions of TRIM47 in cancer pathogenesis and progression, a study recommends that TRIM47 joins in the modulation of ubiquitination-mediated degradation of oncogene products or tumor suppressors (Qian et al. 2022). TRIM47 deletion significantly lessened LPS-induced histological alternation with symptoms of acute lung injury and pulmonary inflammation in TRIM47^−/−^ deficient mice (Herbert 2019). With TRIM47-specific knocking-down and overexpression in endothelial cells, they approved that TRIM47 can significantly inhibit the transcription of numerous pro-inflammatory cytokines, and also reduce monocyte adhesion with HUVECs in cultured systems (Qian et al. 2022). As TRIM47 has a RING domain in possession of a critical role in the ubiquitination of colorectal cancer as an E3 ligase along with promoting tumor occurrence and metastasis, the group suggested that TRIM47 might facilitate the infiltration of tumor cells by ubiquitination tumor-related genes (Wang et al. 2020). Upregulation and overexpression of the ADAR gene occur in over 8% of breast, lung, and hepatic cancers (Liang et al. 2019). Surveys of human cancer-derived cell lines in vitro using RNA interference or gene editing approaches revealed that 11–80% are dependent on ADAR for survival with estimates varying by statistical method and tumor type (Herbert 2019).

Analysis of TCGA data showed that the expression level of TRIM47 was associated with the clinical forecast of TC patients, and the T/N stage of the patients with high expression was higher than that in the patients with low expression. When overexpression of TRIM47, the migration and invasion abilities of FTD-133 cells were significantly enhanced. On the contrary, the TRIM47 knocking-down intervention by lentiviral shRNA decreased the migration and invasion capabilities of THJ-29 T cells. The consequences of the animal model indicated that TRIM47 knocking-down lessened the tumor size of the nude mice. The above evidence exhibits that TRIM47 can promote the malignant formation of TC. Co-IP analysis of the THJ-29 T and FTD-133 showed that the expression of TRIM47 was closely related to ADAR, and immunofluorescence co-localization showed that TRIM47 and ADAR were highly overlapped in the distribution of cellular structures. Subsequently, we approved that ADAR can decrease the stimulating effect of TRIM47 on cell proliferation, sensitization effect against chemotherapeutic drugs, and influence cell viability along with the cooperative effect of TRIM47 on cell migration and invasion.

In pancreatic cancer, a recent study claimed that TRIM47 can accelerate aerobic glycolysis and promote tumor progression by regulating FBP1-associated ubiquitination (Li et al. 2021). They found that TRIM47 expression was significantly upregulated along with the decrease of FBP1 expressions in pancreatic cancer patient tissues linking to a lower survival rate, which was also approved in pancreatic cancer cells as well (Li et al. 2021). It appears that targeting on ubiquitination of the TRIM47/FBP1 axis might develop a novel approach to suppress the worseness of pancreatic cancer (Li et al. 2021). In our study, the results indicated TRIM47 endorses thyroid tumorigenesis via the down-regulation of ADAR*via*TRIM47/ADAR axis as well, which involved GSK-3β mediated phosphorylation. Ubiquitination experiments in vitro showed that TRIM47 can validate ubiquitination in an enzyme activity-dependent manner. As we approved, through binding with GSK-3β protein, TRIM47 can enhance ADAR protein stability and ubiquitination so that TRIM47 endorses ADAR-mediated promotion of tumor migration and survival capability.

Based on our experiments above, we would like to claim that our study suggests a novel theory of ADAR expression modulation by TRIM47 via ADAR-associated ubiquitination pathway along with their possible phosphorylation. These phenomena suggest that a strong potential target for an efficient strategy could be developed with a unique treatment for cohorts with thyroid carcinoma. The novel interaction between TRIM47-ADAR may certainly shed new light in the field of thyroid carcinoma study and inspire future exploration of the potential significance in TC patient treatment.

Through examinations within experiments in vitro and in vivo, we unveiled that TRIM47 significantly suppressed the growth in the clinical samples of thyroid carcinoma, TC tumor cell lines, and xenograft animal models. Further experiments found that TRIM47 interacted with ADAR to facilitate ADAR protein degradation via ubiquitination along with GSK-3β associated phosphorylation on both TRIM47 and ADAR. The stability and regulation of the TRIM47-ADAR-GSK-3β axis could determine the exacerbation of TC tumor expansion, invasion, and metastasis in tumor model animals and patients at high risk of thyroid carcinoma. Our study proposed for the first time that TRIM47-ADAR interaction might serve as a potential target for developing future TC treatment.

Our study found that TRIM47 has a direct interaction with ADAR and can regulate the proliferation, migration, and sensitivity to chemotherapeutic drugs of TC cells by promoting the ubiquitination and degradation of ADAR. Further experimental data showed that this process depends on the activation state of GSK-3β. GSK-3β is a multifunctional serine/threonine kinase that can regulate the fate of its substrates through"initial phosphorylation"(Cheng et al. 2024). We speculate that GSK-3β may phosphorylate ADAR to make ADAR obtain a mark similar to"initial phosphorylation", making it easier for TRIM47 to recognize and catalyze ubiquitination. Since GSK-3β has different activities in different cell backgrounds, its activity state may be different in different subtypes of thyroid cancer. For example, in more aggressive undifferentiated TC, GSK-3β activity is higher, which may further promote the phosphorylation of ADAR and TRIM47-mediated degradation, thereby exacerbating tumor progression. These speculations need further verification.

In this research, we found that TRIM47 promoted the ubiquitination modification of ADAR, leading to its decreased stability. As an E3 ubiquitin ligase, TRIM47 may directly interact with ADAR through its RING finger protein domain to promote its ubiquitination modification. This ubiquitination modification may lead to the degradation of ADAR, thereby affecting its function in the cell. ADAR may edit the transcripts of oncogenes and tumor suppressor genes in tumor cells. We speculate that TRIM47 can affect the ADAR-mediated RNA editing process by regulating the stability of ADAR. The loss of ADAR may lead to reduced RNA editing of key oncogenes, thereby stabilizing β-catenin protein and promoting nuclear translocation. This is consistent with reports that TRIM47 promotes metastasis in liver cancer by activating the Wnt pathway (Tang et al. 2024).

Our study recognizes that various factors may confound our findings. For example, in the clinical sample analysis, patient-related variables such as age, gender, tumor stage, and previous treatment regimens could influence the expression of TRIM47 and other biomarkers. To mitigate these effects, we have performed multivariate regression analyses that adjust for these variables, ensuring that our observed differences are more likely due to the biological factors under investigation rather than extraneous influences. Meanwhile, in our in vitro and in vivo experiments, potential confounders include technical variability (e.g., differences in cell culture conditions, passage number, and batch effects) and biological variability among animal subjects. We addressed these by standardizing experimental procedures, randomizing the assignment of animals to experimental groups, and repeating experiments in triplicate to confirm reproducibility.

However, our research still has some limitations. The clinical sample size in the study was relatively small and only came from one hospital. This study only selected two TC cell lines for in vitro experiments. Although these two cell lines represent different differentiation states of TC to some extent, TC itself has high heterogeneity, and its molecular characteristics, signaling pathway activation status, and sensitivity to treatment may differ significantly among different subtypes.

### Significance

At present, how to successfully control refractory and metastatic diseases is still the basic goal of clinicians to improve the treatment effect of patients with thyroid cancer (TC). More and more clinical proofs indicate that ubiquitin intervention degradation of oncogene products or tumor inhibitors may be related to the etiology of cancer. (TRIM) family proteins take part in a spacious series of cellular procedures, including cell proliferation, differentiation, apoptosis, cell cycle, carcinogenesis, and so on. Most TRIM proteins affect ubiquitin E3 ligase activity and help post-translational modification. Among them, TRIM47 is identified as overexpression in astrocytoma for the first time. In addition, other studies have shown that the expression of TRIM47 is closely related to human glioma and prostate cancer. However, the pathological and clinical role of TRIM47 in TC remains largely elusive. In addition, the imbalance of ADAR expression can lead to cancer, amyotrophic lateral sclerosis, and other diseases, and the pathological and clinical role of ADAR in TC has not been revealed. So far, no studies have reported the complex functions of TRIM47 and ADAR in the occurrence and development of TC. This study discovered that TRIM47 was significantly suppressed in TC clinical samples and tumor cell models. TRIM47 interacted with ADAR to facilitate ADAR protein degradation via ubiquitination, which resulted in the exacerbation of TC tumor expansion, invasion, and metastasis. Thus, such TRIM47-ADAR interaction promises to improve our understanding of TC pathogenesis and might serve as a potential target for future TC treatment development.

## Conclusion

Overall, this study demonstrates that TRIM47 interacted with ADAR to facilitate ADAR protein degradation via ubiquitination, and GSK-3β associated phosphorylation, which might serve as a potential target for developing new TC treatment.

## Data Availability

Raw data are available on https://www.jianguoyun.com/p/DSLC3H4QuaiFChiD8tcFIAA.

## Abbreviations

ADAR: Adenosine deaminases acting on RNA
ASC: American Cancer Society
ATC: Anaplastic thyroid carcinoma
Co-IP: Co-immunoprecipitation
DDP: Diamminedichloroplatinum
DTC: Differentiated thyroid carcinoma
EPI: Epirubicine
EMT: Epithelial-mesenchymal transition
GSK-3β: Glycogen synthase kinase 3β
IHC: Immunohistochemistry
NCT: Non-cancerous tissue
qPCR: Quantitative real-time PCR
shRNA: Short hairpin RNAs
TC: Thyroid carcinoma
TCGA: Cancer Genome Atlas
TNM: Tumor–node–metastasis
TRIM: Tripartite motif
UPP: Ubiquitin-proteasome pathway
UPS: Ubiquitin-proteasome system
